# Suboptimal capability of individual machine learning algorithms in modeling small-scale imbalanced clinical data of local hospital

**DOI:** 10.1371/journal.pone.0298328

**Published:** 2024-02-23

**Authors:** Gang Li, Chenbi Li, Chengli Wang, Zeheng Wang

**Affiliations:** 1 Department of ICU, 3201 Hospital, Hanzhong, Shaanxi, China; 2 Data61, CSIRO, Clayton, VIC, Australia; 3 Manufacturing, CSIRO, West Lindfield, NSW, Australia; The First Hospital of Jilin University, CHINA

## Abstract

In recent years, artificial intelligence (AI) has shown promising applications in various scientific domains, including biochemical analysis research. However, the effectiveness of AI in modeling small-scale, imbalanced datasets remains an open question in such fields. This study explores the capabilities of eight basic AI algorithms, including ridge regression, logistic regression, random forest regression, and others, in modeling a small, imbalanced clinical dataset (total n = 387, class 0 = 27, class 1 = 360) related to the records of the biochemical blood tests from the patients with multiple wasp stings (MWS). Through rigorous evaluation using k-fold cross-validation and comprehensive scoring, we found that none of the models could effectively model the data. Even after fine-tuning the hyperparameters of the best-performing models, the results remained below acceptable thresholds. The study highlights the challenges of applying AI to small-scale datasets with imbalanced groups in biochemical or clinical research and emphasizes the need for novel algorithms tailored to small-scale data. The findings also call for further exploration into techniques such as transfer learning and data augmentation, and they underline the importance of understanding the minimum dataset scale required for effective AI modeling in biochemical contexts.

## 1. Introduction

Due to strong performance in generative and representative modeling for scientific problems, the application of Artificial Intelligence for Science (AI4Science) has become increasingly attractive in many traditional research fields [1–3]. This trend is driven by the unprecedented ability of AI algorithms to uncover complex patterns, make predictions, and generate new insights from vast and intricate datasets. In biochemical and bioinformatical studies particularly, a lot of great works have emerged intensively in recent years: These works encompass a wide range of applications, including drug discovery [4,5], protein structure analysis [6,7], traditional medicines [8–10], genetic sequence analysis [11], disease prediction [12,13], and pandemic management [14], etc. For instance, deep learning techniques have been employed to predict protein structures with remarkable accuracy, revolutionizing the field of structural biology [7]. Similarly, machine learning models like random forest and support vector machines have been instrumental in identifying potential biomarkers for various diseases, thereby aiding in early diagnosis and personalized treatment [11,15]. The convergence of AI technologies with traditional biochemical and bioinformatical methods is thus heralding a new era in scientific exploration and discovery, promising to reshape the landscape of biomedical research in the coming years.

However, many of those AI models require large-scale datasets, or big data, for sufficient training and efficient generalizing [16]. The necessity for vast amounts of data usually stems from the complexity of the models and the intricate relationships they are designed to capture [17]. In the context of biochemical and bioinformatical studies, this often means needing detailed and comprehensive molecular structures, genetic sequences, or clinical records. Therefore, while big data has enabled unprecedented advancements in these fields, it also presents significant challenges. The collection, preprocessing, and management of such extensive datasets can be resource-intensive and time-consuming [17]. Moreover, issues related to data privacy, security, and standardization further complicate the utilization of big data in AI-driven research [18]. The requirement for large-scale data also raises concerns about the applicability of the AI models in scenarios where only limited data are available–this is a very often case in biochemical or clinical research. For the clinicians and researchers in a local or regional hospital with limited patient records, this problem becomes even more severe.

Thus, a pressing question that remains is: what types of AI algorithms can be effectively applied to model small-scale biochemical data? In other words: Is it feasible to directly employ individual basic AI models to achieve satisfactory modeling outcomes? Addressing this question is crucial, as not all research scenarios have access to large datasets. The ability to glean meaningful insights from limited data is vital for expanding the applicability of AI in scientific research. If basic AI models prove inadequate for the task, it would be prudent to exercise caution in relying solely on them. Such shortcomings warrant a deeper investigation into the root causes and may necessitate the development of innovative solutions, such as generative models [15,19,20], transfer learning [21,22], or the use of large language models [23].

To explore this, we adopted a small dataset collected from clinical practice, comprising the biochemical blood test (BBT) results and biophysical conditions of patients with multiple wasp stings (MWS). The size of this dataset is modest (n = 387 after preparation), reflecting a common challenge in specialized medical research. We examined the modeling capability of eight fundamental machine learning algorithms with this small-scale dataset, aiming to assess their performance and identify potential strategies for effective modeling in data-constrained environments of biochemical research.

## 2. Materials and methods

In this study, anonymized BBT results and biophysical conditions from 408 individuals diagnosed with MWS were analyzed, with the data as of May 2023 backing to June 2016 (data accessed in 30^th^ of May 2023). After an initial assessment of the unprocessed data, 21 patient records were omitted due to noticeable mistakes (such as indecipherable content) or improper entries. Within those 21 records, 6 patients were admitted to the hospital as a result of anaphylactic shock from wasp stings. Among them, 4 patients received initial treatment at local clinics near the sting location, and 2 patients were attended to in an ambulance. Since the shock symptoms had ameliorated by the time of hospital admission, these cases were not considered in the comparative analysis.

**Fig 1:**
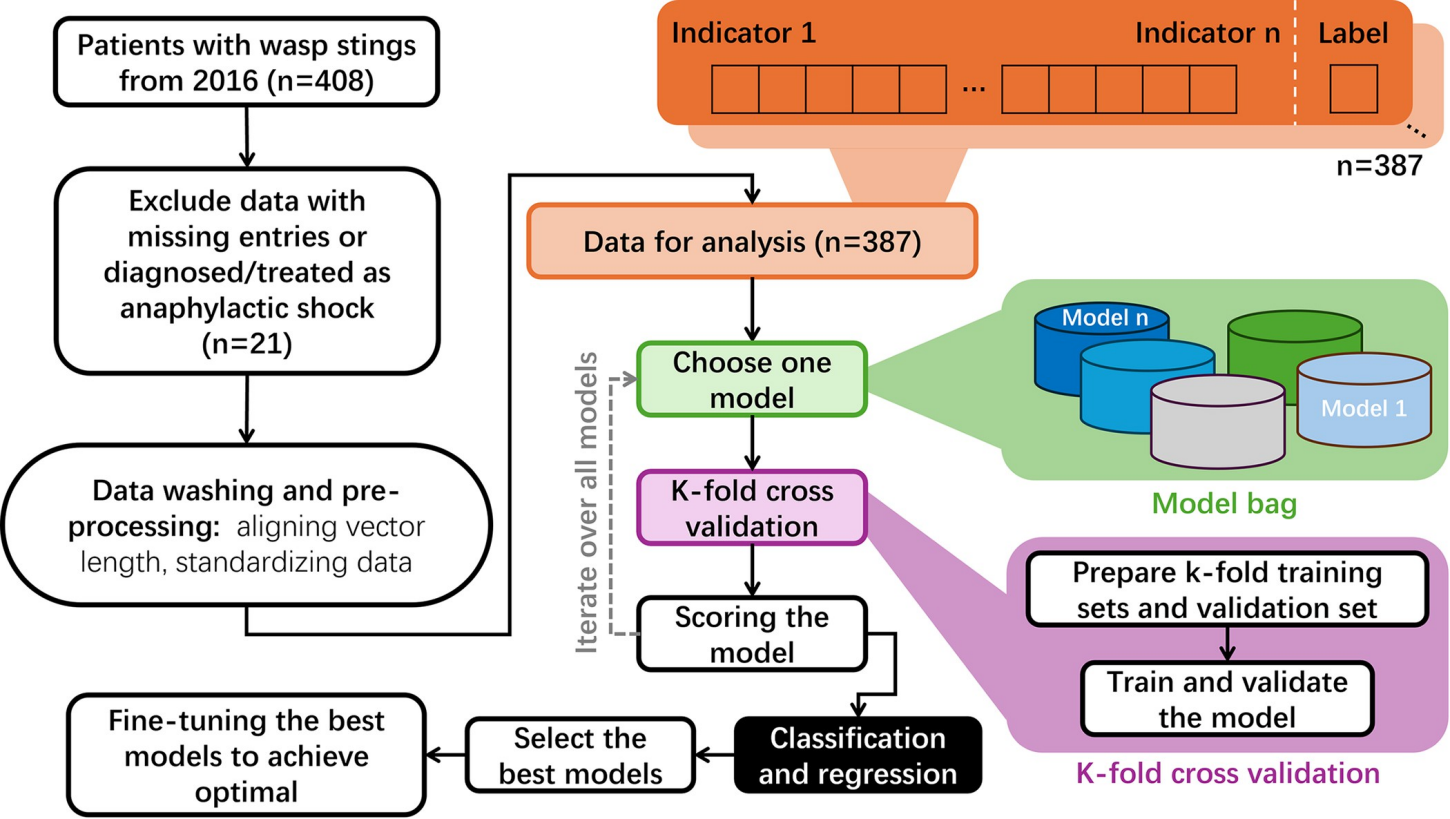
The structure of the datasets. In total 387 records were analyzed in this study, including 27 negative labels and 360 positive labels. Each record contains 18 values of biochemical test results. These 387 were randomly split into training set and validation set with different folds for the training and validation. HGB, hemoglobin; PLT, blood platelet; WBC, white blood cell; ALT, alanine aminotransferase; AST, aspartate aminotransferase; CK, creatine kinase; CK-mb, creatine kinase mb; LDH, lactate dehydrogenase; Myo, myoglobin; PT, prothrombin time; FIB, fibrinogen; APTT, activated partial thromboplastin time; PCT, procalcitonin; IL-6, interleukin-6; Cr, creatinine; Urea, urea; DBIL, direct bilirubin; IBIL, Indirect bilirubin.

Within the dataset, each observation consists of 21 attributes: 18 results from BBT (utilized to assess biochemical conditions, such as ALT, CK, etc.), and 3 physical conditions, specifically the counts of wasp stings on the head, limbs, and trunk. Accompanying these features, a label is assigned to delineate the ultimate clinical outcome (either survival or death). The structure of the dataset and the full names of the BBT items can be found in Fig 1. The objective of the modeling process is to enable AI algorithms to discern the patterns within a subset of these features (the training set) and subsequently predict the label for previously unseen data (the test or validation set).

**Fig 2:**
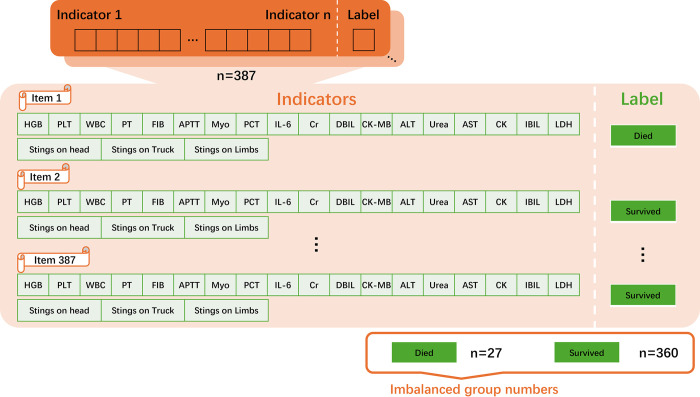
The process of analyzing the clinical biochemical and physical data for evaluating the AI models. After data pre-preparation, the data were fed into one selected model. Each model in the model bag was trained and k-fold-cross validated. Several best-performing models were then fine-tuned to obtain the best capability.

To achieve this goal, the dataset was partitioned into two distinct segments: a training set and a test set. The training set was employed to instruct the models, a process also known as supervised learning, while the test set was reserved for the models to forecast the labels for features not encountered during the training phase. To reduce the uncertainty of randomly choosing the training and test sets, in this process, k-fold cross-validation was implemented to provide a robust evaluation of the models [24]. This technique involves dividing the training data into ’k’ subsets (each subset contains 387/k samples), or folds, and iteratively training the model on ’k-1’ folds while validating on the remaining fold, which means the training set contain 387*(k-1)/k samples. This process ensures a more comprehensive assessment of the model’s performance across different subsets of the data. Following the training and validation stages, the model’s performance was assessed using the Mean Squared Error (MSE), the R2 score, and other scoring matrices. To reflect the imbalanced label categories, stratified sampling was adopted in the k-fold process, where each sub-group data contains the same ratio of minority labels as the whole dataset. The entire workflow of this procedure is depicted in Fig 2.

A total of eight AI regression models were utilized in this study, including ridge regression, logistic regression, random forest (RF) regression, gradient boosting (GB) regression, Gaussian Naïve Bayes (GNB), linear Support Vector Machine (SVM), Radial Basis Function (RBF) kernel SVM, and multi-layer perceptron regression (using artificial neural network, ANN). These models were individually trained and assessed in a systematic manner to identify the best-performing ones. We first used the models to perform a classification task. After evaluating classification performance of the models, we also performed the regression tasks to comprehensively judge the models: we set a threshold (0.5) to convert all calculated probabilities into the classifications. To keep the evaluation fair, no threshold-moving techniques were involved in the current study. Subsequently, the hyperparameters of the selected models were meticulously tuned to further refine and optimize their modeling capabilities. All models were compiled using the Scikit-learn package of Python [25], with default settings (using package-defined hyperparameters).

To fine-tune the models, we identified several key hyperparameters that significantly affect the model’s performance (e.g., the maximum depth of the random forest, which can influence the overfitting of the model), according to the Scikit-learn package. These parameters were then systematically adjusted with the same data while continuously monitoring relevant performance metrics. When multiple hyperparameters were involved, their impact on performance was visualized using color maps. Conversely, when only a single hyperparameter required tuning, we plotted its relationship with the performance metrics using line graphs.

## 3. Results and discussion

A significant characteristic of this dataset is imbalance between the number of positive labels (n = 27) and negative labels (n = 360), with more patients surviving after treatment. This imbalance is reflective of a common scenario in general biochemical research, where results often exhibit an uneven distribution. This label imbalance poses a significant challenge for machine learning algorithms, potentially leading to a model biased towards the majority class, in this case, survivors. Such a bias could undermine the model’s sensitivity in identifying the minority class, which is often the critical group in medical prognosis. Furthermore, the skewed distribution may compromise the model’s generalizability to other patient cohorts with a more balanced outcome distribution. To address these issues, techniques such as oversampling the minority class or employing cost-sensitive learning algorithms are often recommended [26]. However, so far, those methods rely on a certain level of prior knowledge and there’s no discussion about the clinical datasets where introducing any prior knowledge should be much more careful. We will show in the following part that failure to account for label imbalance could result in misleading performance metrics and limit the clinical applicability of the model.

It’s worth noting that the issue extends beyond mere algorithmic bias. In a clinical setting, a model biased towards predicting survival could lead to inadequate resource allocation, as the healthcare system may underestimate the number of patients requiring intensive care. Moreover, the ethical implications of such a bias cannot be ignored, as it could result in unequal treatment of patients based on flawed predictions. We have further considered the real-world implications of label imbalance in clinical settings. For diseases where the survival rate is naturally high or low, it’s crucial that machine learning models are robust enough to handle such imbalances without losing predictive power. This is especially pertinent in resource-constrained healthcare systems where accurate prognosis is vital for effective resource allocation.

**Fig 3:**
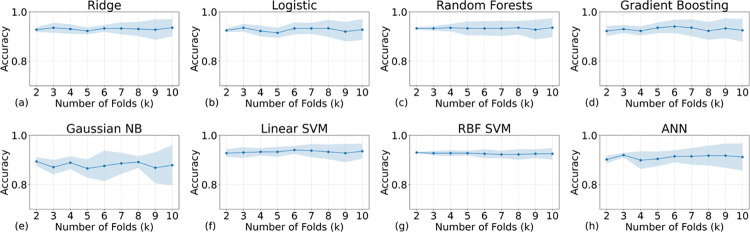
The relationship between the average accuracies (the higher the better) and their variance against the k values for the eight models from (a) ridge regression to (h) ANN. The titles above the subfigures indicate the models’ names. K values can also represent the size of the validation set: The smaller the validation set the higher the K values. All models show a good mean accuracy (>0.8).

We initially evaluated the accuracy of the models using k-fold cross-validation. Fig 3 illustrates the relationship between the average accuracy and its variance (shallow regions) against the k values for the eight models. As depicted in the figure, ridge regression and linear SVM produced the best desirable outcomes, characterized by the highest accuracies. Conversely, GB and ANN demonstrated the worst accuracies, characterized by the highest accuracies and variances. However, all models’ accuracies are higher than 0.8 –a relatively acceptable accuracy value (note that these values are only valid for this datasets). Whereas a model with high accuracy but low specificity may lead to unnecessary treatments or interventions, thereby straining healthcare resources and potentially causing harm to patients.


$$Accuracy=\frac{True\;Positive\;(TP)+True\;Negative\;(TN)}{Total\;Observations}$$ (1)

**Fig 4:**
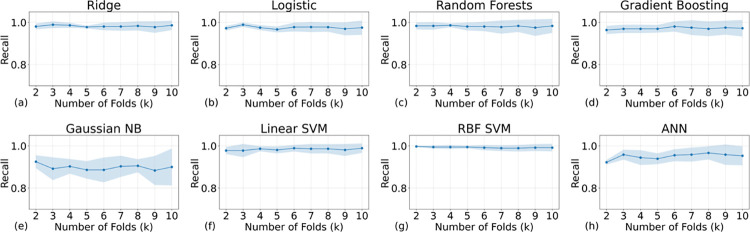
The recalls of all the AI models with different training samples (the higher the better). The titles above the subfigures indicate the models’ names. K values can also represent the size of the validation set: The smaller the validation set the higher the K values. All models show a good mean recall score (>0.8).

**Fig 5:**
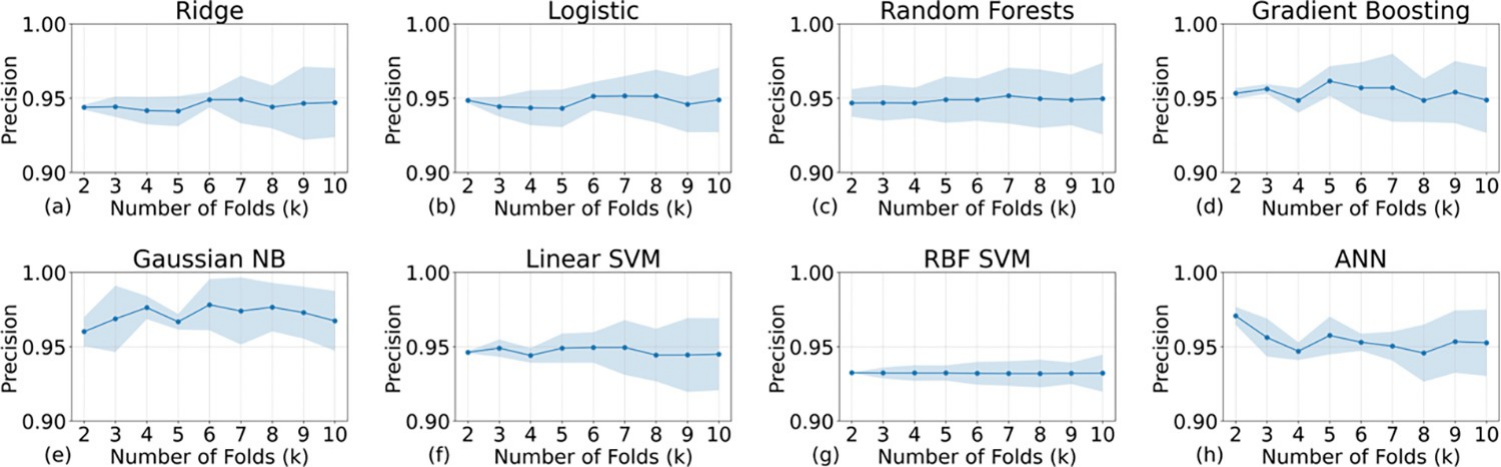
The relationship between the average precisions and their variances against the k values for the eight models (the higher the better). The titles above the subfigures indicate the models’ names. K values can also represent the size of the validation set: The smaller the validation set the higher the K values. All models show a good precision score (>0.9).

**Fig 6:**
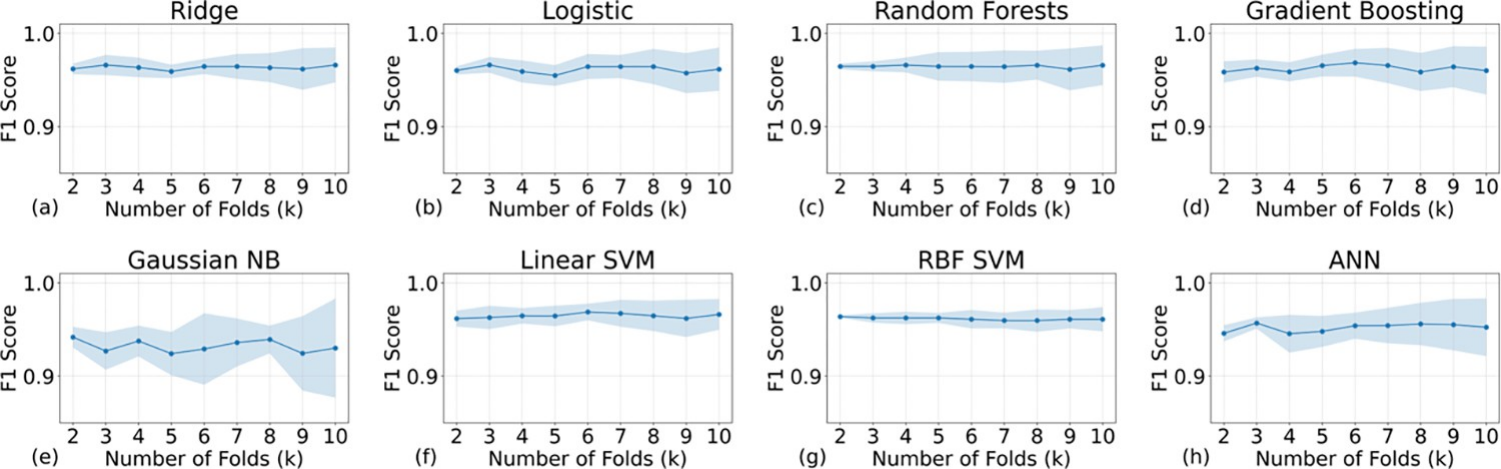
The F1 scores of all the AI models with different training samples (the higher the better). The titles above the subfigures indicate the models’ names. K values can also represent the size of the validation set: the smaller the validation set the higher the K values. All F1 scores are reasonably high because of the high accuracy, precision, and recall.

Figs 4 and 5 present the performance metrics of recall and precision, respectively. Remarkably, all recall values exceed 0.8, while all precision values surpass 0.9, indicating a robust model performance. As delineated by Eqs (1)–(3), these high metrics underscore the model’s exceptional efficacy in accurately classifying positive outcomes. In the context of an imbalanced dataset, achieving high values for both recall and precision is especially indicative of the model’s exceptional ability to correctly identify True Positives (TP). A high recall suggests that the model is proficient at capturing the majority of actual positive cases, thereby minimizing False Negatives (FN). Concurrently, a high precision signifies that among the predicted positive cases, a large proportion are indeed TP, minimizing False Positives (FP), which can also be validated by the F1 scores (see Eqs 1–4 for the definition) that are shown in Fig 6. Therefore, these metrics collectively underscore the model’s precision and reliability specifically in identifying TP cases, which is often the most critical aspect in medical prognosis models.


$$Recall=\frac{True\;Positive\;(TP)}{True\;Positive\;(TP)+False\;Negative\;(FN)}$$ (2)

$$Precision=\frac{True\;Positive\;(TP)}{True\;Positive\;(TP)+False\;Positive\;(FP)}$$ (3)

$$F1\;Score=2\times \frac{Precision+Recall}{Precision\times Recall}$$ (4)

However, in the context of managing patients with MWS, the accurate prediction of True Negatives (TN) takes on heightened significance for optimizing patient outcomes. Accurate TN predictions serve as a crucial decision point for healthcare providers, enabling the targeted allocation of medical resources right from the moment of a patient’s admission. By correctly identifying these TN cases, healthcare systems can proactively deploy interventions, therapies, or additional monitoring, thereby substantially improving the likelihood of patient survival. In essence, a high rate of TN predictions not only conserves valuable healthcare resources but also serves as a pivotal factor in enhancing patient survival rates.

To evaluate the classification capability for the TN, we adopted the specificity score, which can be defined as

$$Specificity=\frac{True\;Negative\;(TN)}{True\;Negative\;(TN)+False\;Positive\;(FP)}$$ (5)

**Fig 7:**
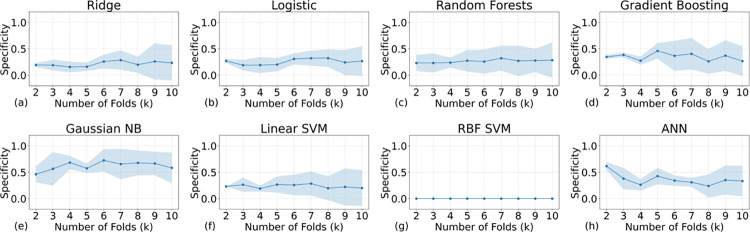
The specificity scores of all the AI models with different training samples (the higher the better). All models’ specifications are lower than the acceptable threshold (normally 0.6, indicating 60% true negative labels can be successfully identified).

**Fig 8:**
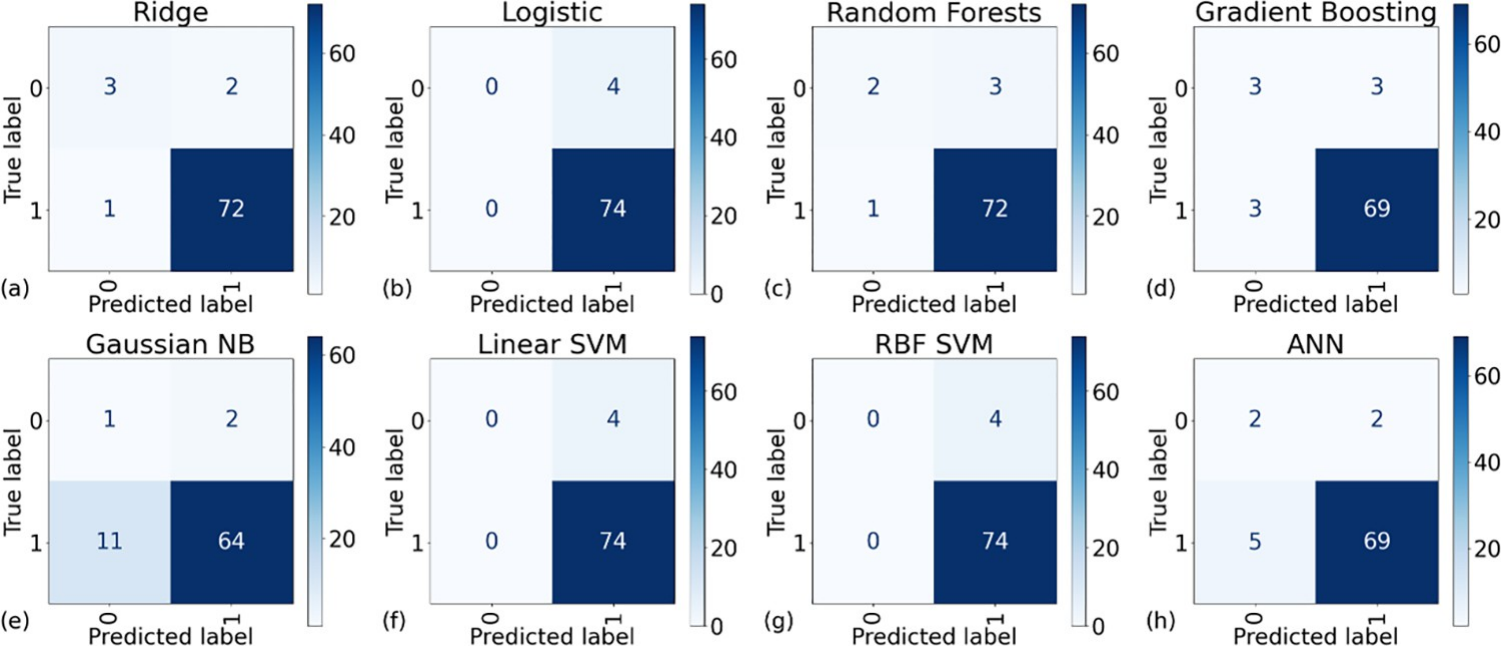
The confusion matrices of all the AI models. Here, the training set n = 309 and the validation set’s n = 78. In this unbalanced small dataset, it is important to focus on the element at the top left corner (true and predicted labels are 0). This element represents the correct classification of the negative labels (TN).

We conducted an evaluation of model specificity employing k-fold cross-validation, the results of which are presented in Fig 7. This figure delineates the relationship between average specificities and their associated variances across varying k-values for the eight models under consideration. Notably, the RBF SVM model exhibited suboptimal performance, manifesting both the lowest specificity (equals 0) and the highest misclassification among the models. In contrast, GNB emerged as the most effective in terms of specificity. However, it’s important to note that even the best-performing model achieved a specificity value of only approximately 0.6. Detailed insights into the classification performance for both TN and TP are readily available in the confusion matrices, displayed in Fig 8. A closer examination reveals a pronounced rate of misclassification for instances with a true label of 0 (Died).

In summary, the elevated values of accuracy, precision, and F1 score observed in our models can be attributed to the models’ propensity to predict most instances as positive labels. Given the scarcity of negative labels in the dataset, the cost of misclassifying them is minimal, leading to inflated values for these metrics. However, the low specificity underscores the models’ limitations in correctly identifying negative cases. These findings collectively highlight the significant impact of label imbalance in small-scale datasets on the performance of machine learning algorithms, cautioning against the sole reliance on commonly used metrics like accuracy, precision, and F1 score for model evaluation.

Having extensively evaluated the performance of various classification models in terms of their ability to accurately identify TP and TN, we also need to evaluate the performance of the regression modeling. The objective of this subsequent analysis is to delve into the continuous variables that may provide a more detailed information of the patient outcomes’ probabilities or patient survival rates, thereby providing a more nuanced understanding of the factors during the healthcare process.

**Fig 9:**
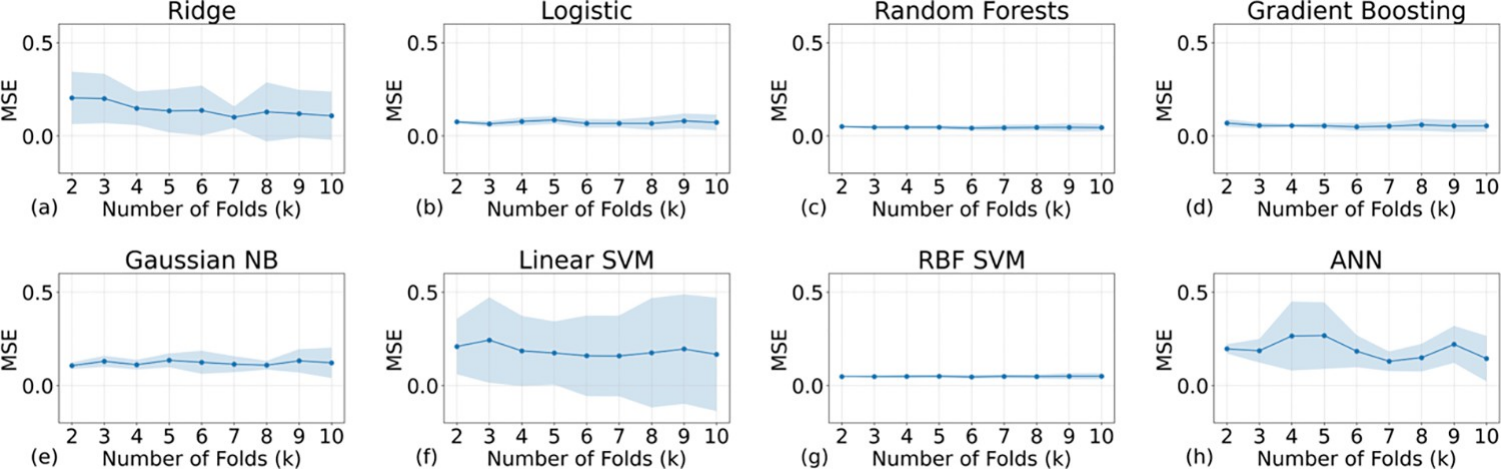
The mean squared errors (MSE) of all the AI models with different training samples. The regression modeling is complementary to the classification. The titles above the subfigures indicate the models’ names. K values can also represent the size of the validation set: The smaller the validation set the higher the K values. All MSEs are below 0.5, which is in accordance with the classification tasks.

**Fig 10:**
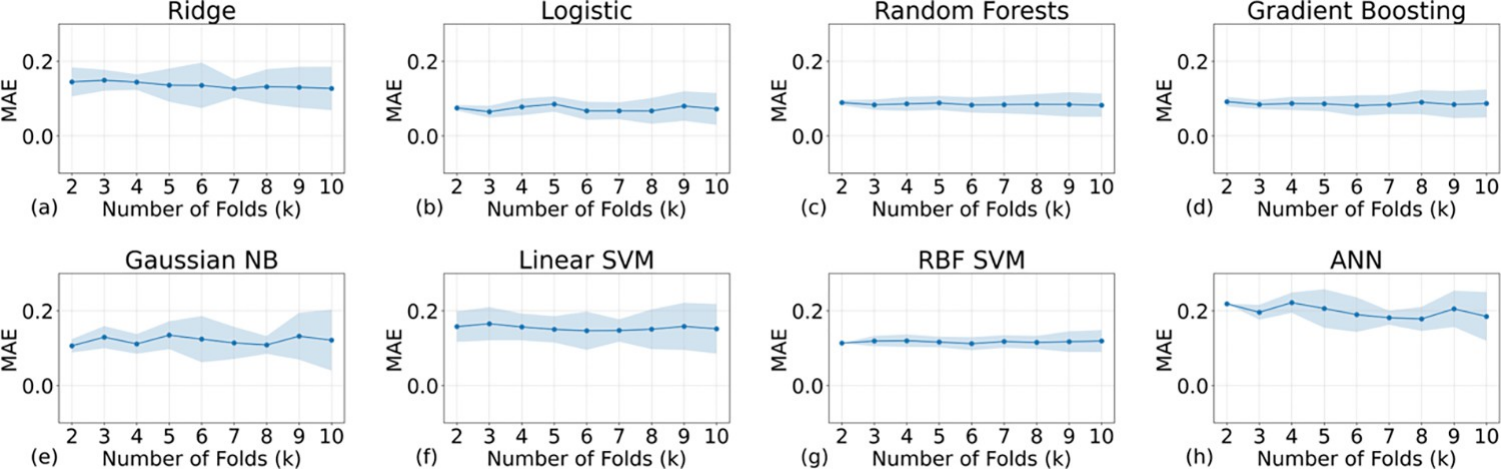
The mean absolute errors (MAE) of all the AI models with different training samples. The regression modeling is complementary to the classification. The titles above the subfigures indicate the models’ names. K values can also represent the size of the validation set: The smaller the validation set the higher the K values.

In assessing the regression performance, we began with the MSE via k-fold cross-validation. Fig 9 plots the average MSE against k values for the eight models, showing ridge regression and linear SVM with the highest MSEs, indicative of suboptimal performance. RF, GB, and RBF SVM, however, registered the lowest MSEs, with RF and GB exhibiting the least variance, suggesting more stable predictions. The similar results can be found in Fig 10, which shows the mean absolute errors (MAE) of each model.

**Fig 11:**
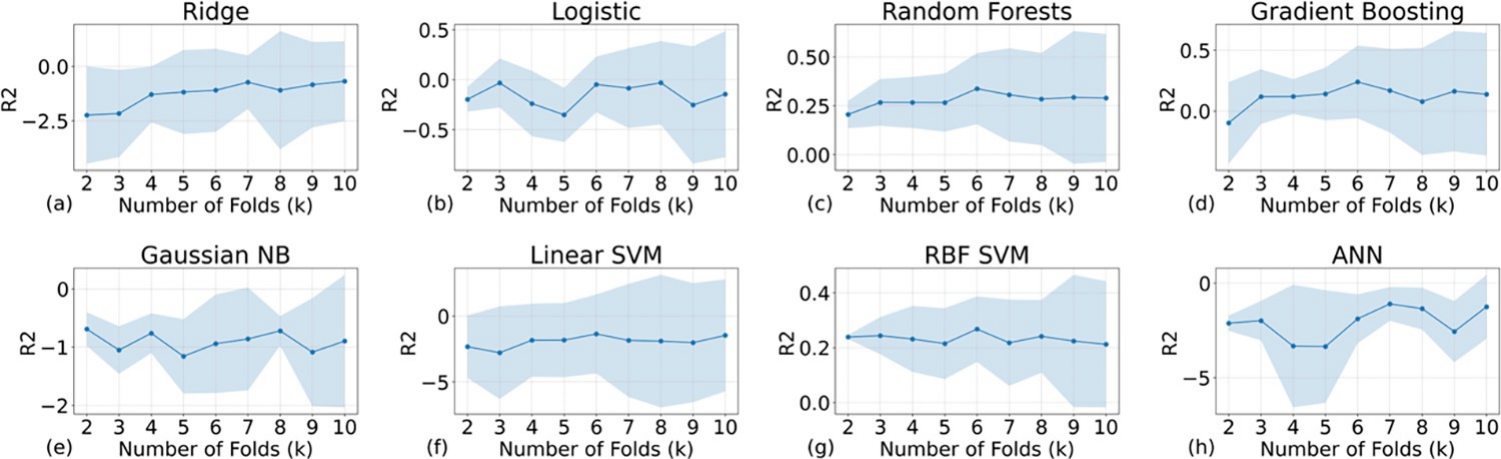
The R2 scores of all the AI models with different training samples. Because the models cannot effectively model the patterns of true negative labels, the R2 scores are below the acceptable threshold (normally 0.5, indicating a much better performance than randomly guessing).

The R2 scores, reflecting the variance in the dependent variable explained by independent variables, were next on our evaluation list. Fig 11 indicates that only RF and RBF SVM models achieved R2 scores above zero, implying a reasonable fit for regression tasks. In contrast, GB and ridge regression, which performed adequately in classification, saw a decline in regression validation.

**Fig 12:**
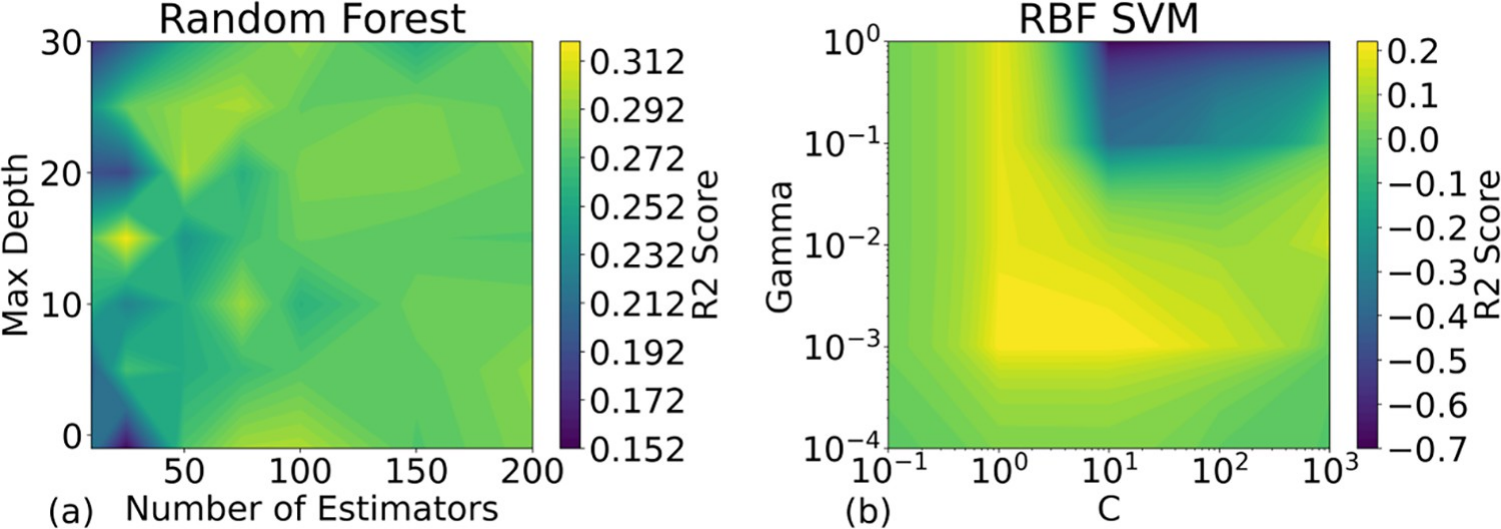
The fine-tuning of the two most promising models regarding the R2 scores. For (a) the Random Forest model, the maximum depth and the number of estimators were tuned. The color map represents the R2 values under each (number of estimators, maximum depth) point. For (b) the RBF SVM, the hyperparameters are Gamma and C.

Hyperparameter tuning for RF and RBF SVM was detailed in Fig 12, targeting key parameters such as max depth, number of estimators, Gamma, and C. Yet, their peak performance did not cross the 0.5 mark, questioning their predictive reliability beyond random chance. This suggests a possible mismatch between model complexity and the nuanced nature of the studied clinical data.

**Fig 13:**
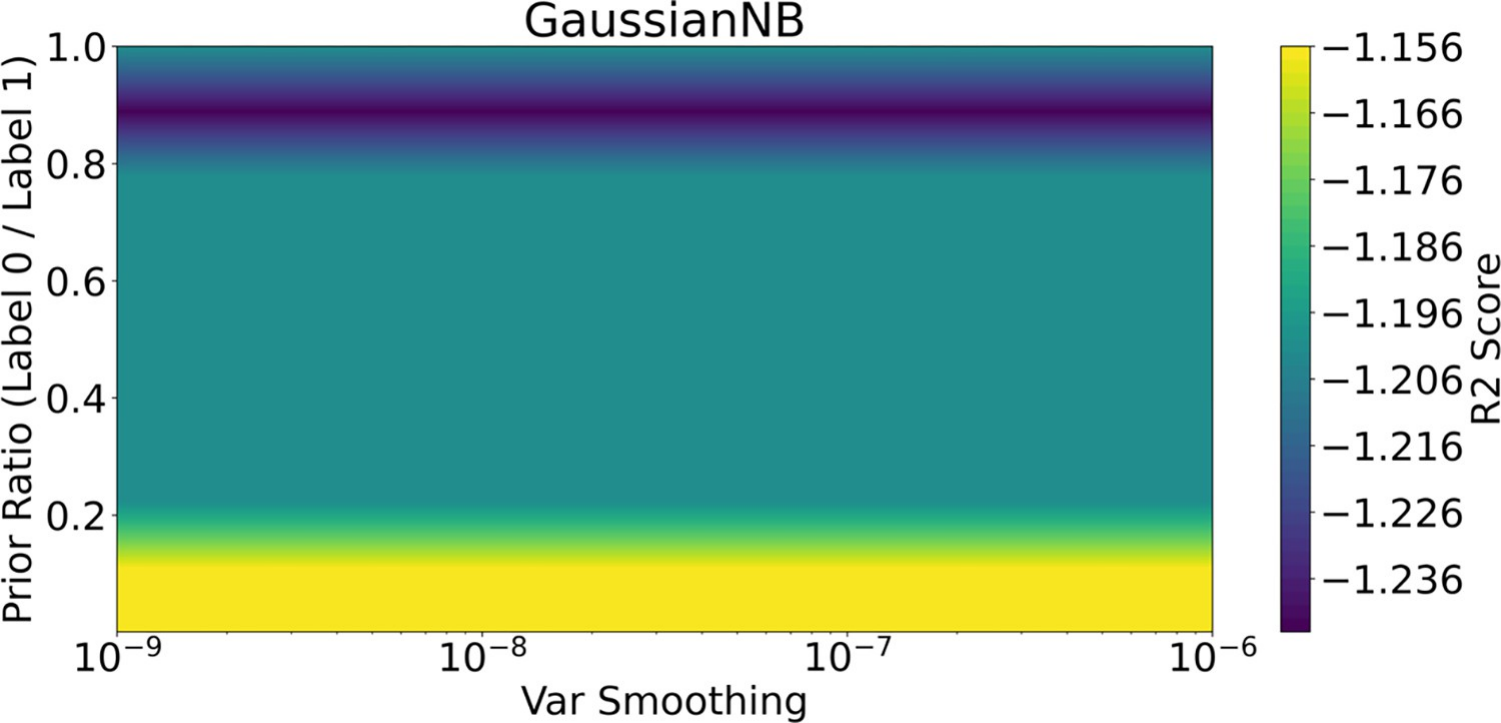
The fine-tuning of the GB model (the one with highest specificity score in k-fold cross validation tests) regarding the R2 scores. Unfortunately, the GB’s R2 is below 0. The hyperparameters for Gaussian NB model are the prior ratio and the var smoothing. By changing these two parameters, different R2 scores can be extracted and shown in the color map.

For the GNB model, despite its classification promise, fine-tuning of prior ratio and variance smoothing did not yield effective modeling of the dataset, as shown in Fig 13. This difficulty with small, imbalanced datasets may stem from the GNB’s sensitivity to the underlying data distribution.

The results suggest that the inherent complexity of the clinical data, possibly due to its multifaceted nature and the imbalance in the dataset, poses significant challenges for modeling. The small sample size and the disproportionate label distribution likely contribute to the models’ inability to generalize effectively, which is a common hurdle in clinical data analysis where outcomes are often skewed towards one class. This underscores the need for more sophisticated or tailored modeling approaches that can handle such data intricacies.

Compared with other studies, although the accuracies of the models in this study match the contemporary studies, the specificity in particular, can only reach values around 0.4, which is below the values of the references [27,28]. However, it should be noted that in the referred studies, large-scale, balanced datasets were used, which is reckoned to be able to benefit the modeling capability.

In the context of this study, the exploration of alternative methodologies may provide avenues for further investigation. Techniques such as transfer learning [29], data augmentation [20,30], and synthetic data generation [31] could potentially be employed to leverage existing data and knowledge for new tasks. Transfer learning, for instance, allows a model trained on one task to be adapted for a second related task, potentially mitigating the need for extensive data in the target domain. Data augmentation techniques, which create variations of the existing data, and synthetic data generation, which creates entirely new data samples, could also enhance the training process.

However, these advanced techniques often still necessitate a certain level of prior knowledge or a sufficient size of the initial dataset for effective implementation. The balance between data sufficiency and model complexity is a nuanced challenge, particularly in the field of biochemical research where data may be imbalanced, scarce, or expensive to obtain for a local regional hospital. Moreover, understanding the boundary of the dataset scale and determining the precise quantity of data required for training an AI model in this specific domain remains an open question. This issue underscores the need for further study, not only to optimize model algorithms but also to contribute to the broader understanding of AI efficiency and effectiveness in general clinical or biochemical research.

This work, while comprehensive, has certain limitations. First, we deliberately refrained from integrating prior knowledge into our modeling process to better mirror real-world scenarios. However, it is generally acknowledged that incorporating prior knowledge can significantly enhance model performance in various tasks. Incorporating clinically-informed prior knowledge, in particular, may prove beneficial in future iterations of this study. Second, our study utilized a single-center dataset, which may inherently carry biases due to regional variations. Such biases could potentially limit the generalizability of our findings.

Furthermore, the field of AI is evolving at an unprecedented pace, with new models and methodologies emerging continuously. This study, by focusing on the evaluation of eight fundamental algorithms, may not encompass the full spectrum of advancements in the field. Such a focus, while providing valuable insights, also presents an inherent limitation. Future research could benefit from exploring a wider array of cutting-edge techniques, such as clinically informed rescaling, to capture the rapidly changing landscape of AI technology more comprehensively.

## 4. Conclusion

In summary, this study employed a small clinical dataset with imbalanced classes, encompassing both biochemical and physical features, to scrutinize the capabilities of eight fundamental AI algorithms in supervised learning tasks. Despite the promising advances in AI, our findings demonstrate that all eight models with default settings failed to effectively model the data in validation processes. Even after fine-tuning the hyperparameters of the best-performing models, the results remained below acceptable thresholds. The results reveal that many AI algorithms, as they stand today, may not be the panacea for all challenges in biochemical research.

These findings prompt a critical reflection on the current state of AI in the field and suggest that more concerted efforts are needed. Future work may focus on preparing large-scale datasets, exploring techniques like transfer learning, data augmentation, or synthetic data generation, or developing novel algorithms specifically tailored to small-scale, imbalanced datasets. The compromise between data sufficiency and model complexity remains a nuanced challenge, and this study contributes insights into the ongoing dialogue surrounding the effective application of AI in biochemical research. The pursuit of these avenues may pave the way for more robust and reliable AI-driven solutions in the field, fostering innovation and enhancing our understanding of complex biochemical phenomena where the dataset’s size is limited and records of the categories are imbalanced.

## References

[1] Clyde A., “AI for science and global citizens,” *Patterns*, vol. 3, no. 2, p. 100446, Feb. 2022, doi: 10.1016/j.patter.2022.100446 35199069 PMC8848001

[2] Johnson R., “Artificial, augmented and automated chemistry,” *Nat*. *Chem*., vol. 13, no. 9, pp. 811–813, Sep. 2021, doi: 10.1038/s41557-021-00779-y 34400818

[3] Clyde A., Ramanathan A., and Stevens R., “Large Language Models for Science,” in *Artificial Intelligence for Science*, WORLD SCIENTIFIC, 2023, pp. 643–669. doi: 10.1142/9789811265679_0034

[4] Paul D., Sanap G., Shenoy S., Kalyane D., Kalia K., and Tekade R. K., “Artificial intelligence in drug discovery and development,” *Drug Discov*. *Today*, vol. 26, no. 1, pp. 80–93, Jan. 2021, doi: 10.1016/j.drudis.2020.10.010 33099022 PMC7577280

[5] Smalley E., “AI-powered drug discovery captures pharma interest,” *Nat*. *Biotechnol*., vol. 35, no. 7, pp. 604–605, Jul. 2017, doi: 10.1038/nbt0717-604 28700560

[6] Jumper J. et al., “Highly accurate protein structure prediction with AlphaFold,” *Nature*, vol. 596, no. 7873, pp. 583–589, Aug. 2021, doi: 10.1038/s41586-021-03819-2 34265844 PMC8371605

[7] Senior A. W. et al., “Improved protein structure prediction using potentials from deep learning,” *Nature*, vol. 577, no. 7792, pp. 706–710, Jan. 2020, doi: 10.1038/s41586-019-1923-7 31942072

[8] Wang Z., Li L., Song M., Yan J., Shi J., and Yao Y., “Evaluating the traditional chinese medicine (TCM) officially recommended in china for COVID-19 using ontology-based side-effect prediction framework (OSPF) and deep learning,” *J*. *Ethnopharmacol*., vol. 272, p. 113957, Feb. 2021, doi: 10.1016/j.jep.2021.113957 33631276 PMC7899032

[9] Wang Z., Li L., Yan J., and Yao Y., “Approaching high-accuracy side effect prediction of traditional chinese medicine compound prescription using network embedding and deep learning,” *IEEE Access*, vol. 8, pp. 82493–82499, 2020, doi: 10.1109/ACCESS.2020.2991750

[10] Yao Y. et al., “An ontology-based artificial intelligence model for medicine side-effect prediction: taking traditional chinese medicine as an example,” *Comput*. *Math*. *Methods Med*., vol. 2019, pp. 1–7, Oct. 2019, doi: 10.1155/2019/8617503 31662790 PMC6791233

[11] Libbrecht M. W. and Noble W. S., “Machine learning applications in genetics and genomics,” *Nat*. *Rev*. *Genet*., vol. 16, no. 6, pp. 321–332, Jun. 2015, doi: 10.1038/nrg3920 25948244 PMC5204302

[12] Peters D. P. C. et al., “Big data–model integration and AI for vector‐borne disease prediction,” *Ecosphere*, vol. 11, no. 6, Jun. 2020, doi: 10.1002/ecs2.3157

[13] Yuan X., Chen J., Zhang K., Wu Y., and Yang T., “A Stable AI-Based Binary and Multiple Class Heart Disease Prediction Model for IoMT,” *IEEE Trans*. *Ind*. *Inform*., vol. 18, no. 3, pp. 2032–2040, Mar. 2022, doi: 10.1109/TII.2021.3098306

[14] Vaishya R., Javaid M., Khan I. H., and Haleem A., “Artificial Intelligence (AI) applications for COVID-19 pandemic,” *Diabetes Metab*. *Syndr*. *Clin*. *Res*. *Rev*., vol. 14, no. 4, pp. 337–339, Jul. 2020, doi: 10.1016/j.dsx.2020.04.012 32305024 PMC7195043

[15] Wang Y., Li C., and Wang Z., “Advancing Precision Medicine: VAE Enhanced Predictions of Pancreatic Cancer Patient Survival in Local Hospital,” *IEEE Access*, vol. 12, pp. 3428–3436, 2024, doi: 10.1109/ACCESS.2023.3348810

[16] Liew S.-L. et al., “A large, open source dataset of stroke anatomical brain images and manual lesion segmentations,” *Sci*. *Data*, vol. 5, no. 1, p. 180011, Feb. 2018, doi: 10.1038/sdata.2018.11 29461514 PMC5819480

[17] Schwartz R., Dodge J., Smith N. A., and Etzioni O., “Green AI,” *Commun*. *ACM*, vol. 63, no. 12, pp. 54–63, Nov. 2020, doi: 10.1145/3381831

[18] Liang W. et al., “Advances, challenges and opportunities in creating data for trustworthy AI,” *Nat*. *Mach*. *Intell*., vol. 4, no. 8, pp. 669–677, Aug. 2022, doi: 10.1038/s42256-022-00516-1

[19] Plesovskaya E. and Ivanov S., “An Empirical Analysis of KDE-based Generative Models on Small Datasets,” *Procedia Comput*. *Sci*., vol. 193, pp. 442–452, Jan. 2021, doi: 10.1016/j.procs.2021.10.046

[20] Wang Z. et al., “Improving Semiconductor Device Modeling for Electronic Design Automation by Machine Learning Techniques,” *IEEE Trans*. *Electron Devices*, pp. 1–9, 2023, doi: 10.1109/TED.2023.3307051

[21] Li Y., Jiang W., Zhang G., and Shu L., “Wind turbine fault diagnosis based on transfer learning and convolutional autoencoder with small-scale data,” *Renew*. *Energy*, vol. 171, pp. 103–115, Jun. 2021, doi: 10.1016/j.renene.2021.01.143

[22] Zhao W., “Research on the deep learning of the small sample data based on transfer learning,” *AIP Conf*. *Proc*., vol. 1864, no. 1, p. 020018, Aug. 2017, doi: 10.1063/1.4992835

[23] van der Lee C., Ferreira T. C., Emmery C., Wiltshire T. J., and Krahmer E., “Neural Data-to-Text Generation Based on Small Datasets: Comparing the Added Value of Two Semi-Supervised Learning Approaches on Top of a Large Language Model,” *Comput*. *Linguist*., pp. 1–58, Aug. 2023, doi: 10.1162/coli_a_00484

[24] Fushiki T., “Estimation of prediction error by using K-fold cross-validation,” *Stat*. *Comput*., vol. 21, no. 2, pp. 137–146, Apr. 2011, doi: 10.1007/s11222-009-9153-8

[25] Pedregosa F. et al., “Scikit-learn: Machine Learning in Python,” 2012, doi: 10.48550/ARXIV.1201.0490

[26] Mohammed R., Rawashdeh J., and Abdullah M., “Machine Learning with Oversampling and Undersampling Techniques: Overview Study and Experimental Results,” in *2020 11th International Conference on Information and Communication Systems (ICICS)*, Irbid, Jordan: IEEE, Apr. 2020, pp. 243–248. doi: 10.1109/ICICS49469.2020.239556

[27] Kong J. et al., “Network-based machine learning approach to predict immunotherapy response in cancer patients,” *Nat*. *Commun*., vol. 13, no. 1, p. 3703, Jun. 2022, doi: 10.1038/s41467-022-31535-6 35764641 PMC9240063

[28] Huang L. et al., “Machine learning of serum metabolic patterns encodes early-stage lung adenocarcinoma,” *Nat*. *Commun*., vol. 11, no. 1, p. 3556, Jul. 2020, doi: 10.1038/s41467-020-17347-6 32678093 PMC7366718

[29] Xiang E. W., Cao B., Hu D. H., and Yang Q., “Bridging Domains Using World Wide Knowledge for Transfer Learning,” *IEEE Trans*. *Knowl*. *Data Eng*., vol. 22, no. 6, pp. 770–783, Jun. 2010, doi: 10.1109/TKDE.2010.31

[30] Arsalidou M., Skuratov N., Khalezov E., Bernstein A., Burnaev E., and Sharaev M., “Effects of age, gender, and hemisphere on cerebrovascular hemodynamics in children and young adults: Developmental scores and machine learning classifiers,” *PLOS ONE*, vol. 17, no. 2, p. e0263106, Feb. 2022, doi: 10.1371/journal.pone.0263106 35120173 PMC8815867

[31] Dahmen J. and Cook D., “SynSys: A Synthetic Data Generation System for Healthcare Applications,” *Sensors*, vol. 19, no. 5, p. 1181, Mar. 2019, doi: 10.3390/s19051181 30857130 PMC6427177

